# A gridded dataset on densities, real estate prices, transport, and land use inside 192 worldwide urban areas

**DOI:** 10.1016/j.dib.2023.108962

**Published:** 2023-02-09

**Authors:** Quentin Lepetit, Vincent Viguié, Charlotte Liotta

**Affiliations:** a Centre International de Recherche sur L'Environnement et le Développement (CIRED), 45bis, *Av* de La Belle Gabrielle, Nogent-sur-Marne F-94736, France; b TU Berlin, Straße des 17. Juni 135, Berlin D-10623, Germany

**Keywords:** Cities, Urban form, Land cover, Urban economics, Real estate, Transportation

## Abstract

This work presents a gridded dataset on real estate and transportation in 192 worldwide urban areas, obtained from the Google Maps API and the web scraping of real estate websites. For each city of the sample, these data have been associated with the corresponding population density and land cover data, extracted from the GHS POP and ESA CCI data respectively, and aggregated on a 1 km resolution grid, allowing for an integrated analysis. This dataset is the first to include spatialized real estate and transportation data in a large sample of cities covering 800 million people in both developed and developing countries. These data can be used as inputs for urban modeling purposes, transport modeling, or between-city comparisons in urban forms and transportation networks, and allow further analyses on e.g. urban sprawl, access to transportation, or equity in housing prices and access to transportation.

**Specifications Table**

**Table:** 

Subject	Economics.

Specific subject area	Urban form, Urban sprawl, Density, Real Estate, Land cover, Transportation studies, Environmental studies, Urban economics.
Type of data	Tables.
How the data were acquired	Transportation data have been obtained through the Google Maps and Baidu APIs, using Python. Real estate data have been obtained from the web scrapping of real estate websites, using Python. Population density data are taken from the GHS-POP dataset [1], and land cover data are taken from the ESA land cover CCI [2]. Data formating and aggregation have been performed with QGIS and R.
Data format	Raw; Analyzed.
Description of data collection	A global sample of 192 cities has been selected to cover cities on all continents, while maximizing their diversity in terms of location, culture, or history. For each city, a 1km-resolution grid encompassing the urban area has been defined, on which all data (population density, land cover, transportation, and real estate), obtained as detailed in the previous sections, have been aggregated.
Data source location	CIRED (center International de Recherche sur l'Environnement et le Développement), Nogent-sur-Marne, France. Data on 192 cities worldwide. Sources of the real estate data and transportation data can be found in table *DataSources.xlsx*, available in the Zenodo repository. Population density and land cover data have been obtained from GHS-POP [1] and ESA Land cover CCI [2].
Data accessibility	Repository name: Zenodo. Data identification number: 6,821,394. Direct URL to data: 10.5281/zenodo.6821394
Related research article	C. Liotta, V. Viguié, Q. Lepetit, Testing the monocentric standard urban model in a global sample of cities, Reg. Sci. Urban Econ. 97 (2022) 103,832. 10.1016/j.regsciurbeco.2022.103832. [3]

**Value of the Data**

• This dataset is the first dataset containing spatialized data on transportation and real estate in a large sample of cities in both developed and developing countries. With the corresponding land cover and density data, it allows working on city structures with a global perspective.
• These data can be used as inputs for urban modeling purposes. In particular, transportation, real estate, population density, and land cover data can be used for urban modeling in urban economics, as urban economics theories rely on the hypothesis that urban structures, and in particular housing supply and prices and population spatial distribution, depend on land use constraints and transportation costs. Transportation and population density data can also be used as inputs for transport modeling.
• These data can be used for between-city comparisons in urban forms and transportation networks, for instance in geographical studies and urban economics, and allow further analyses on e.g. urban sprawl, access to transportation, or equity in housing prices and access to transportation. These data can also be used as examples of differences between cities in urban forms and transportation networks for teaching purposes.
• By suggesting a standardized protocol, we make our dataset expandable to other countries and cities in the world, enabling reuses in transport, environmental or economic studies.

## 1 Data Description

The dataset is composed of four files (stored in both R data format .rds and Comma Separated Values .csv), which share a similar structure: each line corresponds to a pixel in a city. The first columns of each file are the same (Table 1), but the other columns are specific. The files are:

• LandCoverData: Land cover data records (Table 2).

**Table 2:** Land cover data records (LandCoverData).

Variable	Type	Description
‘OpenedToUrb'	[float]	Fraction of the grid cell that is unconstrained, i.e. opened to urbanization.
‘ClosedToUrb'	[float]	Fraction of the grid cell that is constrained, i.e. closed to urbanization.
‘ESACCI10′ to ‘ESACCI230	[float]	ESA CCI land cover data in m² (see file EsacciReclassification.xslx or land cover CCI user guide https://climate.esa.int/media/documents/CCI_Land_Cover_PUG_v2.0.pdf, accessed 08/10/2021).
• PopulationDensityData: Population density data records (Table 3).

**Table 3:** Population density data records (PopulationDensityData).

Variable	Type	Description
‘PopDensitySource'	[string]	Population density source name.
‘PopDensityYear'	[integer]	Population density year.
‘PopDensity'	[float]	Population density inside the corresponding pixel for the corresponding year and population source.
• RealEstateData: Real estate data records (Table 5).
• TransportData: Transport data records (Table 4).

**Table 4:** Transport data records (TransportData).

Variable	Type	Description
‘TransportSource'	[string]	Source of the transport data.
‘RushHour'	[string]	Night rush hour corresponding to the selected transport source and city.
‘TransportYear'	[integer]	Year the transport data were collected.
‘DistanceDriving'	[float]	Distance to the CBD during the city's rush hour, by private car, in meters.
‘DurationDriving'	[float]	Travel time to the CBD during the city's rush hour, by private car, in seconds.
‘DistanceTransit'	[float]	Distance to the CBD during the city's rush hour, by public transportation, in meters.
‘DurationTransit'	[float]	Travel time to the CBD during the city's rush hour, by public transportation, in seconds.

**Table 1:** Common records.

Variable	Type	Description
‘ID'	[integer]	Spatial ID of the grid pixel.
‘X'	[float]	X coordinates of the grid pixel in the city's corresponding UTM projection in meters.
‘Y'	[float]	Y coordinates of the grid pixel in the city's corresponding UTM projection in meters.
‘Area'	[float]	Pixel land area in m².
‘City'	[string]	City name.
‘Country'	[string]	Country name.
‘Continent'	[string]	Continent name.
‘GridEPSG'	[integer]	Spatial Reference System EPSG code of the corresponding city. It gives the spatial projection used for the grid.
‘dCenter'	[float]	Geographical distance to the Central Business District (CBD) in meters.

Table 1, Table 2, Table 3, Table 4, Table 5 present the variables in these files.

**Table 5:** Real estate data records (RealEstateData). Note: suffixes “boxplotOutliers”, “percentilesOutliers” or “hampelOutliers” indicate the outliers’ exclusion method.

Variable	Type	Description
‘Currency'	[string]	ISO 4217 currency code of the corresponding country.
‘TransactionType'	[string]	Type of the transaction of corresponding observation. It can be 'Rent' or 'Sale'.
‘TransactionSource'	[string]	Name of the data source.
‘TransactionMonth'	[string]	Month the data were collected.
‘TransactionYear'	[integer]	Year the data were collected.
‘avgSize'	[float]	Average size of dwellings inside the corresponding pixel, for the corresponding transaction, source, and date, in m².
‘avgPriceSqm'	[float]	Average price per m² of dwellings inside the corresponding pixel, for the corresponding transaction, source, and date, in local currency.
‘medSize'	[float]	Median size of dwellings inside the corresponding pixel, for the corresponding transaction, source, and date, in m².
‘medPriceSqm'	[float]	The median price per m² of dwellings inside the corresponding pixel, for the corresponding transaction, source, and date, in local currency.
RegPriceSqm	[float]	Price per m² of dwellings inside the corresponding pixel, for the corresponding transaction, source, and date, in local currency, obtained by regressing prices on dwelling sizes in the pixel.
‘nRealEstateData'	[integer]	Number of observations inside the corresponding pixel, for the corresponding transaction, source, and date.

We provide two additional tables:

• *DataSources.xlsx* displays the detailed sources and collection dates of transport and real estate data, including the transport data source (column “Transport Data Source”), the rush hour at which the transport data have been collected (column “Rush Hour”), the real estate website from which the real estate data have been collected (column “Real Estate Website”), and the real estate data scrapping date (column “RE data scrapping date”).
• *EsacciReclassification.xslx* displays the nomenclature of the ESA CCI land cover data (column “ESA CCI land cover category”) and how these land cover data have been reclassified between “Opened to urbanization” and “Closed to urbanization” (column “Reclassification”).

## 2 Materials and Methods

### 2.1 Methods

**Fig. 1:**
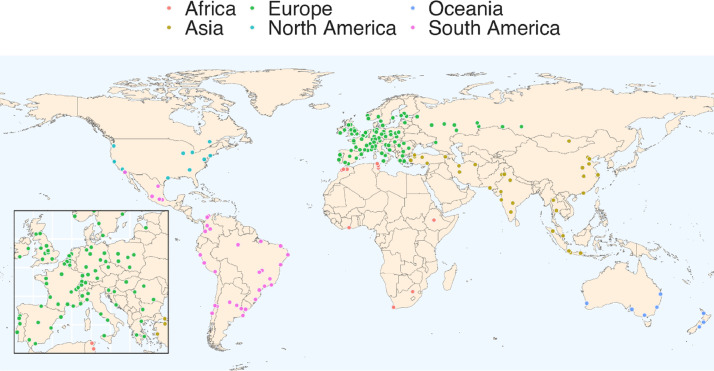
Final sample (192 cities). This figure is taken from the related research article Liotta et al. (2022) [3].

*Selection of the urban areas.* We have chosen the cities of our database following two criteria. First, we selected medium to large cities (with a population of over 300 000) in order to get a large share of the global urban population. Second, we selected cities of different cultural and historical backgrounds and tried to get a wide geographical coverage. In this way, we defined a first sample of 281 cities. However, data collection was possible in 192 cities of this sample (Fig. 1), mainly due to the availability of real estate data, as detailed in the next sections. Figure 4, in the supplementary material, presents the original 281 cities.

**Fig. 2:**
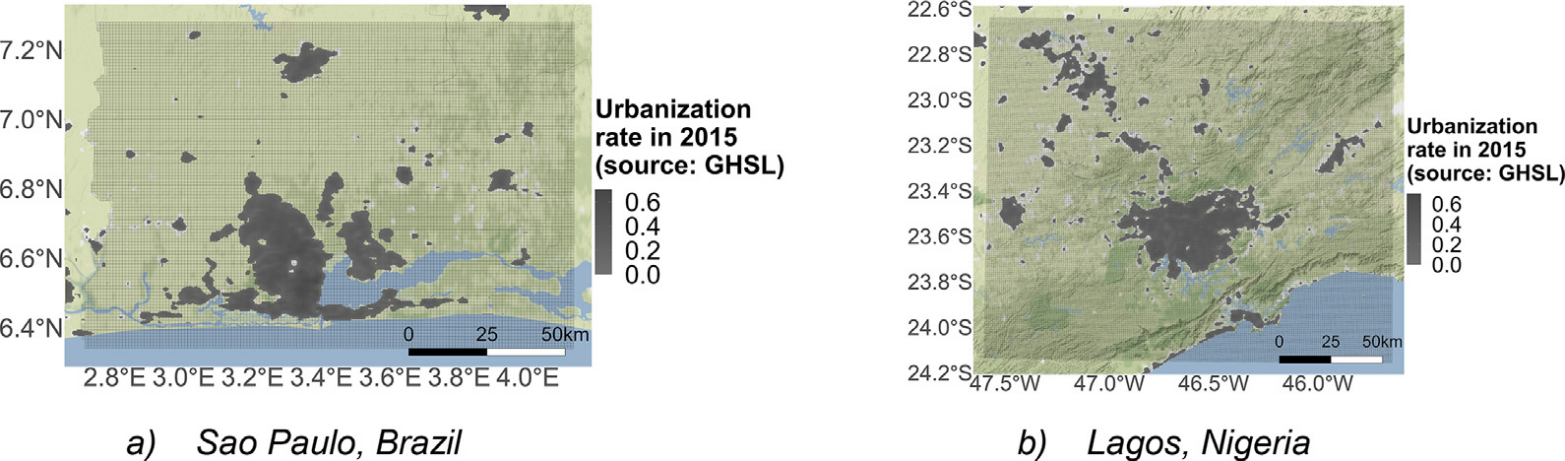
Example of spatial analysis grids.

*Grid analysis.* For each city, we designed a georeferenced grid of a 1 km^2^ resolution, encompassing the whole urban area (Fig. 2). We used these grids to aggregate land cover, population density, real estate, and transportation data at the same resolution.

*Land cover.* We used the European Space Agency land cover data, available worldwide at a 300 m spatial resolution on an annual basis from 1992 to 2015 [2]. These data allow to identify urbanized areas, as well as to distinguish constrained areas, i.e. locations where housing construction is impossible or difficult (water bodies,…) from unconstrained areas (see table *EsacciReclassification.xlsx* in the Zenodo repository).

*Population density.* We used the GHS-POP layers of the open and free GHSL (Global Human Settlement Layer) data of the European Commission, providing population counts per grid cell at a 250 m resolution worldwide and available for 1975, 1990, 2000 and 2015 [1,4].

*Real estate.* We have collected real estate data on rents, sale prices, and dwelling sizes by web scrapping real estate websites from 2017 to 2020. We selected these websites following four criteria:

• the website must have a nationwide coverage to ensure consistent results in each country,
• it must geolocalize the dwellings,
• it must have values for both rent or sale prices and dwelling sizes, and
• it has to be written in the local language and to propose prices in local currency to limit real estate ads targeting expatriates.

Real estate websites and scrapping dates can be found in table *DataSources.xlsx*, available in the Zenodo repository. Then, we aggregated the data and displayed them at the pixel level:

• we aggregated dwelling sizes by computing the mean and the median of data per pixel.
• we compute rents or sale prices per sqm by dividing total rents or sale prices by dwelling sizes, and then display the mean and the median per pixel. Alternatively, we regress total rents or sale prices by dwelling sizes for each pixel to find an estimate of rents or sale prices per sqm for each pixel.
• as a robustness check, we also display these data applying three outliers’ exclusion methods on prices per sqm before the aggregation: boxplot (with a 1.5 coefficient), percentiles (excluding the top and bottom 2.5% values), and hampel.

*Transportation.* We collected transport distances and durations to the city centers using Google Maps and Baidu Maps APIs (Application Programming Interfaces). Different methods have been used in the urban economics literature to identify city centers. Most rely on job density data [5], [6], [7], [8], [9], which are unfortunately not available on a consistent basis in our sample of cities. Therefore, we defined city centers by a compromise between five qualitative criteria: the geographical center of the data, the historical center of the cities, the location of public transports hubs, the official central business district, and the city hall location.

We collected transport data from the centers defined above to each grid cell at typical afternoon rush hours^1^. We collected, when available, both driving and public transport data. Transport data sources and rush hours can be found in table *DataSources.xlsx*, available in the Zenodo repository. It was not possible to collect transport data from each grid cell, so we collected data from 10% of all cells^2^, and then interpolated them using the *interpp* function from R package *akima*.^3^

Code availability. Detailed code generating the database can be accessed from the source code hosted via Github at https://github.com/CIRED/gridded_dataset_192_cities.

### 2.2 Technical Validation

*Land cover.* For land cover, we rely on the validation performed in the ESA CCI technical report [2]. Validating ESA CCI land cover data against GlobCover 2009 data, the authors found an overall accuracy of 71.45%, with the highest accuracies for rainfed cropland, irrigated cropland, broadleaved evergreen forest, and urban areas. The report mentions that other validation tests are undergoing.

*Population density.* For population density, we rely on the existing validations of the GHSL data. Validation tests of the GHSL built-up data, from which the GHSL population data are directly derived, are presented in the JRC technical report [11]. In this report, the GHSL built-up data are validated using two sources: a systematic field survey managed by EUROSTAT and a set of digital cartographic products with building footprints at a scale of 1:10,000 or better gathered from data portals of official websites of public governmental institutions. From comparing GHSL data with these two sources, total accuracies are 0.9628 and 0.8996 respectively. Other validation tests have been conducted by independent studies; for instance, comparing the GHSL built-up data with Baidu data in 20 Chinese cities, Liu et al. (2020) [12] find an R2 of 0.76.

The GHSL population data are more difficult to validate due to the lack of independent and comparable data. However, benchmarking using the GEOSTAT 2011 resident population data on 18 European countries, Freire et al. (2016) [4] found a correlation of 0.83.

*Real estate.* The quality of real estate prices, rents and dwelling size data differs from one city to another. A first source of error comes from the quantity of data that we have been able to collect. To assess the quality of the real estate data, we computed two variables at the city level: the market data cover, as the total population of the city divided by the number of ads, and the spatial data cover, as the number of pixels for which we have real estate data divided by the number of inhabited pixels. For market data cover, we found that in 95 cities out of 192, we have scrapped more than 1 rent ad per 1000 inhabitants, and that in 174 cities out of 192, we have scrapped more than 1 rent ad per 10,000 inhabitants. For spatial data cover, we found that in 109 cities out of 192, the spatial data cover of rent ads is above 10%, and that in 153 cities out of 192, the spatial data cover is above 5%. We report in our data the number of ads per pixel.

A second potential source of mistake is the systemic bias coming from our data sources. The websites that we scrapped present ads, which may not necessarily reflect the actual rents or prices, if margins of negotiations exist, for instance. They may also be biased and present only dwellings that are not representative of the actual dwelling stock. This may especially be the case if, in a city, online ad websites are not the main way of buying or renting a dwelling. To mitigate these risks, we tried to make sure that the websites we scrapped are actually used by locals.

**Fig. 3:**
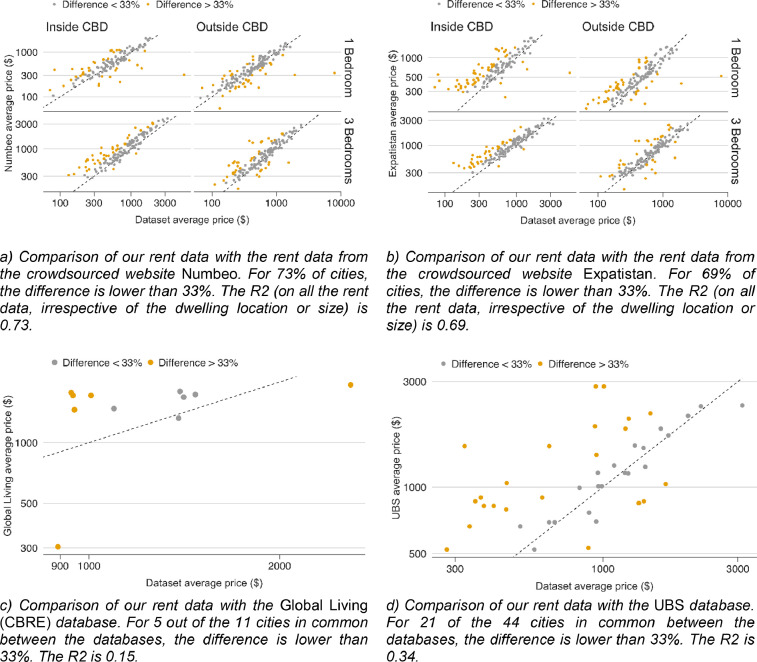
Comparison of our database on rents with external databases for 2019.

To assess these two risks, we checked the validity of our rents and real estate prices against 4 external databases providing averages per city. We used two crowd-sourced websites aiming at describing the cost of living across the world for expatriates, Numbeo [13] and Expatistan [14], to estimate rents and property prices around the world. We also used two databases built for real estate investors: UBS "Prices and Earnings" database [15], and data from CBRE, an expert in real estate and services based in London, whose data were obtained from various local sources [16]. When averaged over the cities, or over the inner core and the outer core of the cities, our data broadly agree with these data (Fig. 3).

*Transportation.* Overall, Google maps and Baidu maps data have several advantages compared with other transport data. Google maps data have a wide coverage compared with other data sources such as OpenStreetMap [19] and are regularly updated. Furthermore, they allow for measuring travel times and distances with and without congestion [20]. For these reasons, they have been used in recent research papers to compute travel times and distances [21].

However, the quality of Baidu and Google maps data is difficult to assess, as these websites use closed algorithms based on users' travel data [17,18]. It should be expected that the quality of these data is a function of the number of users in each city, and should therefore be higher in developed country cities and in large population cities [22], [23], [24]. The quality of travel data for public transport depends on whether transport authorities have shared their data with Google or Baidu. Another source of error comes from the number of data points that we use to measure transport times, and from the interpolation process. This source of error is also difficult to assess. We tried to mitigate it by using grid points close to each other near the center of the city, and further apart from each other when moving away from the center.

## Ethics Statement

• Terms of Service (ToS) and Copyright:
  • GHSL data are provided free of charge. Reuse is authorized, provided the source is acknowledged. Copyright notice: © European Union, 2022
  • The ESA CCI land cover data products are made available to the public by ESA and the consortium and can be used for scientific purposes without any fee on the condition that ESA Climate Change Initiative and its Land Cover project are credited. Copyright notice: © ESA Climate Change Initiative - Land Cover led by UCLouvain (2017).
  • At the time of data collection, the Terms of Service of the scrapped real estate websites were unclear regarding scrapping. However, UK,^4^ France,^5^ and European Union^6^ legislation policies allow for data collection through web scrapping and use for non-profit research regardless of the ToS. The only shared data are aggregated data on dwelling sizes and rents or prices per 1km^2^ grid cell so that raw data are not shared.
  • Google Maps allow for data collection with an API: https://cloud.google.com/maps-platform/terms.
• *Privacy:* All data have been anonymized by a spatial aggregation at a 1 km resolution, preventing from going back to the raw data.
• *Scrapping policy:* Scraping policies of the real estate websites were unclear at the time of the study. However, we limited ourselves to retrieving the dwelling sizes, when available, and rent or prices, and we avoided web scraping of websites employing any measures to limit/block scrapping.

## CRediT authorship contribution statement

**Quentin Lepetit:** Conceptualization, Methodology, Investigation, Software, Validation, Formal analysis, Data curation, Writing – original draft, Visualization. **Vincent Viguié:** Conceptualization, Methodology, Writing – review & editing, Supervision, Funding acquisition. **Charlotte Liotta:** Formal analysis, Data curation, Writing – review & editing, Visualization.

## Declaration of Competing Interest

The authors declare that they have no known competing financial interests or personal relationships that could have appeared to influence the work reported in this paper.

## Data Availability

A gridded dataset on densities, real estate prices, transport, and land use inside 192 worldwide urban areas (Original data) (Zenodo).

## Footnotes

1 We have identified rush hours for each city by extracting a sample of transportation data at 16h, 16h30, 17h, 17h30, 18h, 18h30, 19h, 19h30, and 20h, by comparing the average delay due to congestion, and by defining rush hours as the moment when the average delay is the higher.

2 With a method close to Saiz and Wang (2021) [10], we defined a star shape with 8 branches, centered on the city center, and we collected data from the grid cells at a regular distance on each star branch so that the total number of grid cells for which data are collected sums to 10% of the total number of grid cells.

3 https://cran.r-project.org/web/packages/akima/index.html

## Footnotes

4 https://blogs.lse.ac.uk/impactofsocialsciences/2014/06/04/the-right-to-read-is-the-right-to-mine-tdm/ and https://www.gov.uk/guidance/exceptions-to-copyright, accessed on 29/9/2022.

5 https://urldefense.com/v3/__https://www.culture.gouv.fr/content/download/281379/file/CSPLA*20-*20Annexes-contributions*20-*20rapport*20exceptions*20fouille*20de*20textes*20et*20de*20donn**Aes.pdf?inLanguage=fre-FR__;JSUlJSUlJSUlJSXDqQ!!NLFGqXoFfo8MMQ!79QRDGeuwrwv1ks5QSugxP8OiKbgN537Z3UiNgIhihDsaG16ce2XuHejW9QANfPAapg$, accessed on 29/9/2022.

6 https://eur-lex.europa.eu/legal-content/EN/TXT/HTML/?uri=CELEX:32019L0790&from=FR#d1e961-92-1, accessed on 29/9/2022.
